# Multimodal therapeutic options for esophageal perforations—a single-center experience

**DOI:** 10.3389/fsurg.2025.1662261

**Published:** 2025-10-01

**Authors:** Maximilian Gruber, Lars Kollmann, Johan Friso Lock, Sven Flemming, Stanislaus Reimer, Markus Brand, Armin Wiegering, Alexander Meining, Ivan Aleksic, Christoph-Thomas Germer, Florian Seyfried

**Affiliations:** 1 Department of General-, Visceral-, Transplantation-, Vascular-, and Pediatric Surgery, University Hospital Wuerzburg, Wuerzburg, Germany; 2 Department of Gastroenterology, Caritas Hospital, Bad Mergentheim, Germany; 3 Department of Gastroenterology, University Hospital Wuerzburg, Wuerzburg, Germany; 4 Department of General, Visceral, Transplant, and Thoracic Surgery, University Hospital of Frankfurt, Frankfurt, Germany; 5 Department of Thoracic and Cardiovascular Surgery, University Hospital Wuerzburg, Wuerzburg, Germany

**Keywords:** esophageal perforation, Boerhaave syndrome, EndoVAC, esophagectomy, EVT

## Abstract

**Background:**

Esophageal perforation is a life-threatening condition with a high mortality rate. The current therapeutic options range from conservative to endoscopic to surgical treatment. We aimed to compare specific patterns of multimodal management of spontaneous vs. other esophageal perforations.

**Methods:**

The data from all consecutive patients diagnosed with either spontaneous (Boerhaave syndrome, BS) vs. other esophageal perforation (OEP) between 2010 and 2023 were prospectively collected and retrospectively analyzed. The primary endpoint was in-hospital mortality. The secondary endpoints were overall complications (Comprehensive Complication Index, CCI), therapy-associated complications, oral nutrition at discharge, and length-of-stay.

**Results:**

In total, 32 patients were identified, of whom 15 were diagnosed with BS and 17 with OEP. Initially, 11/32 (34.4%) were primarily treated endoscopically, 12/32 (37.8%) with surgery, and 8/32 (25.0%) with a combined treatment. Patients with BS had larger perforations (22.50 vs. 15.00 mm, *p* = .05) and higher complication scores (CCI: 61.80 vs. 45.60, *p* = .076). Over the course, the primary therapeutic regimen (endoscopic or local surgical treatment) had to be escalated in 36.4% of the patients. Overall, the in-hospital mortality rate was 9.4% (3/32 patients), with a strong trend toward a higher mortality rate in patients with BS (20.0 vs. 0.0%, *p* = .053). Diagnoses of BS and sepsis at admission (*β* = 28.387, *p* = .012) were independent risk factors for a higher CCI score.

**Conclusions:**

BS and sepsis at admission are risk factors for a complicated course. Endoscopy is the first choice for diagnosis and initial treatment. Patients with mediastinal gross contamination or large defects usually need surgical intervention, which should not be delayed.

## Introduction

Esophageal perforations are rare, occurring in approximately 3.1/1,000,000 individuals per year, but are potentially life-threatening situations with a mortality rate of up to 50% (1–3). Thereby, esophageal perforations can be classified into barotrauma due to excessive vomiting (Boerhaave syndrome, BS) or other—mostly iatrogenic—reasons (other esophageal perforations, OEP). Therapeutic algorithms are provided by the World Society of Emergency Surgery (WSES) guidelines for esophageal emergencies (4), with a variety of different approaches, ranging from endoscopic interventions [stent, over-the-scope-clip (OTSC), endoscopic vacuum therapy (EVT), VacStent GI^TM^, etc.] to either surgical local treatment with primary defect closure and fundoplication or salvage esophagectomy (5–8). However, the overall evidence is limited as most studies that conclude that individualized treatment strategies should be based on distinct clinical scenarios and timing describe small case series (5–7).

The aim of the present study was to compare our interdisciplinary and multimodal treatment regimens and outcomes for the management of BS and OEP with a special focus on safety, success rate, and complications.

## Material and methods

The data from all consecutive patients who were diagnosed and treated for esophageal perforation—either BS or OEP—between 2010 and 2023 at the University Hospital, Wuerzburg, Germany, were collected in a prospective database and retrospectively analyzed. The primary endpoint was in-hospital mortality. The secondary endpoints were overall complications (Comprehensive Complication Index, CCI), therapy-associated complications, oral nutrition at discharge, and length-of-stay (LOS).

### Interdisciplinary treatment

Clinical management was led by an interprofessional team (an experienced endoscopist, an upper-gastrointestinal (GI) surgeon, an intensive care professional, a thoracic surgeon, and specialized nursing staff) with experience in all treatment options. After the diagnosis was confirmed, primary endoscopic treatment (stent or EVT), surgery (fundoplication or esophagectomy), or a combined approach was performed based on clinical parameters, including the perforation's location and size, mediastinal contamination, the duration between the injury and diagnosis, and the patient's overall condition. The baseline medical treatment included the avoidance of all oral intake, volume replacement, broad-spectrum antibiotic treatment, and parenteral or enteral (Jejunofix) feeding.

### Role of the nursing staff

Our nursing staff is routinely trained once a year on the management of patients with complex esophageal diseases. This particularly includes the expected clinical courses and possible aberrations. When clinical warning symptoms (fever, pulmonary distress, shock, etc.) occur, drainage secretions are conspicuous, or the EndoVAC systems do not work, the surgeon on call or upper-GI surgeon is contacted.

### Endoscopic procedures

The endoscopic approaches for managing esophageal perforation include the placement of fully covered esophageal stents and EVT. OTSCs were not routinely used in this patient cohort.

Briefly, stent application was carried out either in the endoscopy unit or in the intensive care unit (ICU). Fully covered self-expanding metal stents (FC-SEMS) with a shaft diameter of 23–25 mm were used. Prior to SEMS application, endoscopic cleansing of the leakage cavity was performed, if possible. After inserting a wire into the stomach, the SEMS was introduced. The positioning of the proximal edge of the SEMS and the release of the SEMS were carried out under endoscopic control and confirmed by x-ray.

The EVT procedures were performed under propofol sedation or general anesthesia (9). The sponge used was an Eso SPONGE (B. Braun Melsungen AG, Melsungen, Germany), with a gastric feeding tube and absorbent wound dressing (Suprasorb CNP Drainage Film; Lohmann & Rauscher International, Rengsdorf, Germany). First, the perforation and (if applicable) any extraluminal cavity were cleaned. Patients with defects large enough for endoscope passage and large extraluminal cavities received intracavitary EVT and patients with small defects received intraluminal therapy. In some cases, intracavitary and intraluminal therapies were combined.

The sponge was adequately positioned under endoscopic visualization before a negative pressure of −125 mmHg was applied. The frequency of sponge exchanges usually varied from 3 to 6 days, depending on the defect and clinical appearance. The treatment strategy for esophageal and/or upper-GI EVT treatment has been published elsewhere (9).

### Laparoscopic transhiatal defect closure and fundoplication

All the surgeries were performed using a minimally invasive approach. After establishing the capnoperitoneum and port placement, removal of contaminated fluid from the abdomen and mediastinum was performed. Under clean conditions, the hiatus was explored and mediastinal adhesiolysis with simultaneous intraoperative endoscopy was performed. This was followed by laparoscopic suturing of the esophageal defect (full-thickness) and coverage using Nissen or Dor fundoplication. Finally, a transhiatal drain was placed. In some cases, combined treatment with preemptive EVT was performed.

### Salvage esophagectomy

Primary salvage esophagectomy was performed in patients with large defects and gross contamination when endoscopic or local surgical treatment was considered insufficient. In these cases, debridement of the pleural cavity and mediastinum, along with esophagectomy with cervical esophagostomy and feeding gastrostomy, was performed. Secondary salvage esophagectomy was performed upon interdisciplinary discussion when endoscopic or local surgical options did not lead to sufficient healing of the defect. After esophagectomy, secondary reconstruction was performed in eligible patients after 12–26 weeks.

### Statistical analysis

All statistical analyses were performed using IBM SPSS Statistics 29 (International Business Machines Corporation, Armonk, NY, USA). Descriptive data are reported as means with standard deviations, unless otherwise stated. Comparisons between the analyzed cohorts were performed using the chi-square test, Fisher's exact test, or Mann–Whitney *U*-test. For further risk analysis, a multivariate linear regression analysis was performed. The level of statistical significance was <0.05 (two-sided).

## Results

### Baseline characteristics, establishment of diagnosis, and status at initial referral

Overall, 32 patients were identified during the study period, with 15 diagnosed with BS and 17 with OEP (Table 1).

**Table 1:** Patient characteristics, diagnosis, and perforation details.

Patient characteristics	BS, *n* = 15	OEP, *n* = 17	*P*-value
Baseline characteristics			
Female/male	6/9 (40.0/60.0)	7/10 (41.2/58.8)	0.946
Age years (range)	66.2 (52.0–79.6)	57.0 (18.0–83.4)	0.109
BMI (kg/m^2^)	25.7 (17.6–32.2)	25.8 (22.0–36.5)	0.280
ASA ≥ 3	13 (86.7)	9 (52.9)	0.004
CCS (range)	3.0 (1.0–5.0)	2.0 (0.0–6.0)	0.146
Comorbidities			
Cardiovascular	9 (60.0)	11 (64.7)	0.784
COPD	2 (13.3)	2 (11.8)	0.893
CKD (GFR < 50 ml/min)	2 (13.3)	1 (5.9)	0.471
Liver fibrosis/cirrhosis	0 (.0)	1 (5.9)	0.340
Diabetes mellitus	2 (13.3)	0 (.0)	0.120
Confirmation of diagnosis			
CT scan	2/15 (13.3)	4/17 (23.5)	0.461
Endoscopy	3/15 (20.0)	7/17 (41.2)	0.197
Combined	10/15 (66.7)	6/17 (35.3)	0.077
Size of perforation			
Median in mm (range)	22.5 (6.0–60.0)	15.0 (2.0–50.0)	0.050
small (<10 mm)	1/15 (6.7)	4/17 (23.5)	0.119
intermediate (10–19 mm)	3/15 (20.0)	5/17 (29.4)	0.344
large (≥20 mm)	11/15 (73.3)	5/17 (29.4)	0.042
Unknown	0	3/17 (17.6)	–
Localization of perforation			
Cardia	12/15 (80.0)	15/17 (88.2)	0.522
Cardia and distal esophagus	3/15 (20.0)	1/17 (5.9)	0.228
Cervical	0	1/17 (5.9)	0.340
Reason of perforation			
Functional upper-GI surgery	–	13/17 (76.5)	–
Endoscopy	–	2/17 (11.8)	–
Bolus impaction	–	1/17 (5.9)	–
Aortic stent implantation	–	1/17 (5.9)	–

BS, Boerhaave syndrome; CCS, Charlson comorbidity score; COPD, chronic obstructive pulmonary disease; CKD, chronic kidney disease; GFP, glomerular filtration rate; OEP, other esophageal perforation. Metric data are given in median with range.

The diagnosis was established by endoscopy, a CT scan, or a combination of both. A combination of the two was carried out more often in patients with BS (66.7 vs. 35.3%, *p* = .077). These patients also had larger perforations at the initial endoscopy (22.50 mm vs. 15.00 mm, *p* = .05). Details on the location and reason for the OEP are provided in Table 1.

Overall, the majority (19/32, 59.4%) of the patients were secondarily referred to our tertiary center. This was more frequent in the BS group compared to the OEP group [12/15 (80.0%) vs. 7/17 (41.2%), *p* = .026]. Of the secondarily referred patients, 10/19 (52.6%) initially received antibiotics, while endoscopic or surgical treatment was performed in 3/19 (15.8%), with no differences between the groups. Sepsis at admission was present in 16/32 (34.4%) overall (Table 2).

**Table 2:** Admission data.

Admission	BS, *n* = 15	OEP, *n* = 17	*P* value
Inpatient admission			
Prim. referred	3/15 (20.0)	10/17 (58.8)	.026
Sec. referred	12/15 (80.0)	7/17 (41.2)	
State at admission			
Sepsis	8/15 (53.3)	8/17 (23.5)	.484
Intubation	5/15 (33.3)	1/17 (5.9)	.147
External treatment in secondarily referred patients			
Antibiotic therapy	8/12 (66.7)	2/7 (28.6)	.109
Endoscopic intervention	1/12 (8.3)	1/7 (5.9)	.732
Surgery	1/12 (8.3)	0/7 (.0)	.412
None	2/12 (16.7)	4/7 (57.14)	.030
Initial level of care			
ICU	14/15 (93.3)	13/17 (41.2)	.190
IMC	1/15 (6.7)	1/17 (5.9)	.927
General ward	0 (.0)	3/17 (17.6)	.087

BS, Boerhaave syndrome; ICU, intensive care unit; IMC, intermediate care unit; OEP, other esophageal perforation; Prim., primary; Sec., secondary. Metric data are given on median with range.

### In-house treatment

One patient [1/32 (3.1%)] received conservative primary therapy with a gastric tube and antibiotics, but received local surgical treatment with hiatal exploration, defect suturing, and fundoplication within days due to clinical deterioration.

Endoscopic primary therapy was performed in 11/32 (34.4%) patients. Of these, 8/11 (72.7%) received EVT, with the remaining three patients receiving endoscopic stent placement. Closure using an OTSC was not tried due to the poor tissue conditions.

A total of 4/11 (36.4%) patients needed early escalation of their therapeutic regimen in this group, with two receiving fundoplication and two receiving salvage esophagectomy as a secondary treatment.

Surgery as the primary treatment was performed in 12/32 (37.8%) cases; 8/12 (66.7%) received defect closure and fundoplication. Defect closure and fundoplication were performed less often in patients with BS compared to those with OEP (42.9% vs. 100.0%, *p* = .038). Salvage esophagectomy as the primary treatment was performed in 4/12 (33.3%) patients. The patients with BS had a significantly higher risk for primary or secondary salvage esophagectomy than the patients with OEP (57.1% vs. 0.0%, *p* = .038).

Endoscopic treatment and fundoplication, as a combined primary therapy, were performed in 8/32 (25.0%) cases. Five of the patients who received primary combined therapy had EVT + fundoplication [5/8 (62.5%)], and three in the primary combined therapy group had a combination of endoscopic stent placement + fundoplication [3/8 (37.5%)]. Escalation to salvage esophagectomy as a secondary treatment was needed in 2/8 (25%) of the cases with a combined primary treatment regimen, with a significantly higher risk for patients with BS vs. those with OEP (66.7% vs. 0.0%, *p* = .035).

Additional therapy for pleural contamination was needed more frequently in the patients with BS [thoracic drain: 86.7% vs. 17.6%, *p* < .001, video-assisted thoracoscopy (VATS): 33.3% vs. 0.0%, *p* = .010] (Table 3).

**Table 3:** Treatment data.

Patients course	BS, *n* = 15	OEP, *n* = 17	*P*-value
Time event-therapy <24h	6/15 (40.0)	5/17 (29.4)	0.576
Prim. conservative therapy	1/15 (6.7)	0/17 (0.0)	0.279
Sec. fundoplication	1/1 (100.0)	0/0 (0.0)	–
Prim. endoscopy	4/15 (26.7)	7/17 (41.2)	0.388
EVT	3/4 (75.0)	5/7 (71.4)	0.898
Stent	1/4 (25.0)	2/7 (28.6)	0.898
Altered therapeutic regime	2/4 (50.0)	2/7 (28.6)	0.477
Sec. fundoplication	1/4 (25.0)	1/7 (14.3)	0.658
Sec. esophagectomy	1/4 (25.0)	1/7 (14.3)	0.658
Prim. surgery	7/15 (46.7)	5/17 (29.4)	0.314
Defect closure and fundoplication	3/7 (42.9)	5/5 (100.0)	0.038
Prim. salvage esophagectomy	4/7 (57.1)	0/5 (0.0)	0.038
Altered therapeutic regime	1/7 (14.3)	2/5 (40.0)	0.310
Sec. endoscopic treatment	1/7 (14.3)	2/5 (40.0)	0.310
Sec. salvage esophagectomy	0/7 (0.0)	0/5 (0.0)	–
Prim. combined	3/15 (20.0)	5/17 (29.4)	0.539
EVT + surgery	3/3 (100.0)	2/5 (40.0)	0.090
Stent + surgery	0/3 (0.0)	3/5 (60.0)	0.090
Escalated to sec. esophagectomy	2/3 (66.7)	0/5 (>0.0)	0.035
Additional therapy for pleural contamination	13/15 (86.7)	3/17 (17.6)	<0.001
Thoracic drain	13/15 (86.7)	3/17 (17.6)	<0.001
VATS	5/15 (33.3)	0/17 (0.0)	0.010
ICU			
Days on ICU (range)	20.0 (1–58)	2.0 (0–65)	0.022
Ventilation on ICU; *n* (%)	13/15 (86.7)	12/17 (70.6)	0.052
Duration of ventilation median days (min-max)	7.0 (1–47)	1.0 (0–60)	<0.001
Tracheostomy	3/15 (20.0)	2 (11.8)	0.522
Discharge with Tracheostomy	3/3 (100)	1 (5.9)	0.171
Jejunal feeding tube	10/15 (66.7)	5/17 (29.4)	0.035
Complications			
New onset of sepsis	6/15 (40.0)	0 (0.0)	0.004
Clavien–Dindo classification ≥3b	10/15 (66.7)	6/17 (35.3)	0.077
CCI (range)	61.8 (20.9–100.0)	45.6 (20.9–80.7)	0.076
In-house mortality^a^	3/15 (20.0)	0 (.0)	0.053
Length of stay (days) (range)	24.0 (5–92)	18.0 (8.0–62.0)	0.216
Oral food intake at discharge	7/15 (46.7)	15/17 (88.2)	0.011

BS, Boerhaave syndrome; EVT, EndoVAC therapy; OEP, other esophageal perforation; Prim., primary; Sec., secondary; VATS, video-assisted thoracoscopy. Metric data are presented as median and range.

a 1× pulmonary failure; 1× cerebral bleeding, 1× septic multiorgan failure.

### Patients' outcome

Overall mortality was 3/32 (9.4%), with a strong trend toward higher mortality in the patients diagnosed with BS (20.0% vs. 0.0%, *p* = .053). Patients died due to septic multiorgan failure, pulmonary complications, or cerebral bleeding (Table 3).

Overall, the mean CCI score was 50.05 (20.9 −100.0), with higher CCI scores in the patients diagnosed with BS [61.8 (20.9–100.0) vs. OEP 45.6 (20.9–80.7), *p* = .076].

The patients with BS required a longer ICU stay (20.0 vs. 2.0 days, *p* = .022). Consistently, the duration of ventilation was substantially longer for the patients with BS (7.0 vs. 1.0 days, *p* < .001).

New-onset sepsis was more frequent in the patients with BS (40.0 vs. 0.0%, *p* = .004). Details are shown in Table 3.

### Subgroup analysis of patients with salvage esophagectomy

A total of 8/32 (25.0%) patients needed either primary or secondary esophagectomy, with a higher risk in the patients with BS (46.7% vs. 5.9%, *p* = .008). All three deceased patients suffered from BS. Of the patients who survived after salvage esophagectomy, 3/5 (60%) received secondary reconstruction (retrosternal colon interposition or gastric pull-up) with cervical anastomosis (Supplementary Table S2). Of the non-reconstructed patients, one died of advanced pancreatic cancer and one had a too-low performance status for secondary reconstruction (Supplementary Table S2).

### Univariate and multivariate risk analyses of CCI score after esophageal perforation

Diagnoses of BS and symptoms of sepsis showed a significant linear regression with CCI in the univariate linear regression analysis. In the multivariate analysis, sepsis at admission was identified as an independent risk factor for a higher CCI score (*β* = 28.387, *p* = .012). A diagnosis of BS showed a strong association with higher CCI scores in the multivariate analysis (*β* = 22.441, *p* = .078) (Table 4).

**Table 4:** Univariate and multivariate risk analyses for CCI.

Independent risk factors	Univariate model			Multivariate model		
	*β*	±SD	*P*-value	*β*	±SD	*P*-value
CCI	45.3	±5.5	<0.001	38.0	±11.6	0.005
Time from event to therapy >24 h	4.4	±9.1	0.626	10.0	±10.7	0.365
BS	17.3	±8.4	0.047	22.4	±12.0	0.078
Size of perforation	0.4	±0.3	0.173	−0.4	±0.4	0.325
Sepsis at admission	27.7	±8.7	0.004	28.4	±10.1	0.012

BS, Boerhaave syndrome; CCI, comprehensive complication index; OEP, other transmural esophageal perforation; SD, standard deviation. Metric data are presented as median and range.

## Discussion

Given the current available literature on esophageal perforations, our study, which included 32 patients, represents one of the largest single-center investigations with a direct comparison between BS and OEP and a focus on different distinct treatment patterns (surgery and endoscopy).

A meta-analysis including 559 patients published in 2004 attempted to answer the question of whether it is clinically relevant to distinguish between BS and OEP (10). Here, it has been shown that the mortality rate for patients with BS was 36% while the mortality rate for patients with OEP was significantly lower (19%) (10).

Moreover, various therapeutic approaches are available, ranging from endoscopic interventions to salvage esophagectomy (6–8, 11). In our cohort, 34.4% of the patients received endoscopic treatment (stent or EVT) as their primary therapy, while 37.8% received surgery and 25.0% a combination of surgery and endoscopic treatment. The decision on the primary treatment approach is an interdisciplinary decision.

However, the primary applied therapeutic regime had to be escalated in 36.4% of patients from primary endoscopic treatment to surgery, in 25.0% from primary surgical treatment to additional endoscopic intervention, and in 25.0% from a primary combined therapy to secondary esophagectomy. This emphasizes the necessity of evaluating all cases in a close-knit interdisciplinary manner to identify the best available therapy alternatives according to each patient's individual course as described before (10).

In recent years, there has been an increasing emphasis on endoscopic strategies for leaks and perforations in the upper gastrointestinal tract, specifically, a stent, OTSC, EVT, and VacStent GI^TM^ (7, 8, 12–15).

A recently published retrospective multicenter study has shown success rates of up to 89% for EVT therapy in patients with transmural esophageal perforations (8). However, a closer look revealed that while EVT success was 100% in patients with OEP, the success rate was reduced to 67% when patients with BS were included. In patients with BS, the success rate was only 33% compared to 88% in patients with OEP (8). These results are in line with our data showing that patients with BS treated by EVT have a higher need for additional/escalating therapy, such as surgical defect closure and fundoplication or even salvage esophagectomy.

The patients with BS also tended to have delayed treatment (>24 h), which has been shown to be an independent risk factor for poor prognosis (13, 16–18). We were unable to confirm this in the multivariate analysis, which could be explained by the overall low number of patients. A diagnosis of BS, however, was strongly associated with a higher risk for a complicated course in the multivariate analysis. Additionally, the severity of the mediastinal and/or pleural contamination in the patients with BS was reflected by a VATS rate of 33.3% compared to 0.0% in the patients with OEP (*p* = .01). Sepsis at admission was also identified as an independent risk factor for further complications over the course. This could explain the lower success rate of primary endoscopic treatment for the patients with BS. Furthermore, it is important to note that endoscopic procedures are also associated with a significant risk of complications (12). The recently published data from Kooij et al. show a complication rate of up to 37% for endoscopic treatment of esophageal perforations (19). The main complications in endoscopic stent placement are bleeding, migration, and leakage (19). EVT appears to be advantageous due to a lower migration rate and the combination of closure and drainage (20).

In the event of a contained esophageal perforation, laparoscopic transhiatal debridement, defect closure, coverage with fundoplication, and drainage are a valuable esophagus-preserving option (13, 21). This method is limited whenever the size of the leak exceeds the mobility of the fundus, as this makes sufficient coverage of the defect impossible.

In the context of local closure, we also considered simultaneous preemptive EVT in some of our patients, which was initially described in the context of esophageal cancer resections (22, 23). In the context of BS, there is a significant risk of additional esophagectomy (66.7 vs. 0.0%, *p* = .035) in comparison to OEP, most likely due to the reasons mentioned above.

There is an ongoing debate regarding the use of esophagectomy as a therapeutic approach, but it is still considered a valuable salvage strategy in difficult situations when other options are not available or fail (8). Our policy is to rather act early than to risk destabilization of the patient, as we mentioned before (9, 24). In our study, secondary salvage esophagectomy was more frequently performed in the patients with BS. Given the significant deterioration in prognosis when sufficient therapy is delayed, esophagectomy must be considered, especially in septic patients with large perforations (≥20 mm) and mediastinitis (7, 25), as BS, if left untreated, has a mortality rate of over 90% (26). In particular, as we and previous reports have shown, esophageal discontinuity is not the end of the patient's path as secondary reconstruction can be performed safely at a later stage (27, 28).

Not every patient with BS is treated primarily in a tertiary care center, as they can be referred to a local care center with limited resources and expertise. Based on our own and published data, we recommend primary endoscopic treatment in this situation. In the event of risk factors or clinical deterioration, the patient should be transferred to a center with designated esophageal expertise. In critical patients or the event of deterioration during ongoing EVT/stent therapy, surgery should be considered at an early stage (29, 30).

This study has several limitations. These primarily include the retrospective nature of the study and its relatively low sample size due to the rare occurrence of esophageal perforations. Furthermore, the cases were collected over a relatively long time period (12 years). Moreover, some parameters were only assessed to a limited extent. For example, time-to-treatment must be interpreted with caution, as a relevant proportion of patients were referred after initial treatment and therefore our access to external data was limited.

## Conclusion

Patients with BS often received delayed treatment and had inferior outcomes compared to patients with OEP. Diagnoses of BS and sepsis at admission were independent risk factors for a complicated further course.

Endoscopy is the first choice for diagnosis and initial treatment. Patients with mediastinal gross contamination or large defects usually need surgical intervention, which should not be delayed. Larger perforations, especially with mediastinitis, often need surgical intervention. In elderly and critically ill patients, preconditioning with EVT prior to surgery can be useful. Salvage esophagectomy can be life-preserving and should not be delayed if deemed necessary.

## Data Availability

The original contributions presented in the study are included in the article/Supplementary Material, further inquiries can be directed to the corresponding author.
