# Supplementary material for: Multimodal therapeutic options for esophageal perforations—a single-center experience
Source: Front Surg. 2025 Oct 1;12:1662261. doi: 10.3389/fsurg.2025.1662261 (PMC12521197; doi:10.3389/fsurg.2025.1662261)
Supplement: Supplementary file 1 [file Table1.docx]

Supplement Table 1: State of admission for primary and secondary referred patients

|  | BS  n=15 | OEP  n=17 | P value |
| --- | --- | --- | --- |
| **Prim. referred patients** | 3/15 (20.0) | 10/17 (58.8) | .700 |
| Sepsis | 1/3 (33.3) | 1/10 (10.0) | .673 |
| Intubation | 1/3 (33.3) | 0 (0.0) | .168 |
| ICU | 3/3 (100.0) | 8/10 (80.0) | .400 |
| IMC | 0 | 0 | - |
| General ward | 0/3 (0.0) | 2/10 (20.0) | .147 |
| **Sec. referred patients** | 12/15 (80.0) | 7/17 (41.2) | .026 |
| Sepsis | 6/12 (50.0) | 3/7 (42.9) | .764 |
| Intubation | 4/12 (33.3) | 1/7 (14.3) | .457 |
| ICU | 11/12 (91.7) | 5/7 (71.4) | .243 |
| IMC | 1/12 (8.3) | 1/7 (14.3) | .596 |
| General ward | 0/12 (0.0) | 1/7 (14.3) | .146 |

BS=Boerhaave syndrome, ICU=Intensive Care Unit, IMC=Intermediate Care Unit, OEP=other esophageal perforation, Prim.=primary, Sec.=secondary. Metric data are given in median with range.
