# Supplementary material for: Multimodal therapeutic options for esophageal perforations—a single-center experience
Source: Front Surg. 2025 Oct 1;12:1662261. doi: 10.3389/fsurg.2025.1662261 (PMC12521197; doi:10.3389/fsurg.2025.1662261)
Supplement: Supplementary file 2 [file Table2.docx]

Supplement Table 2: Subgroup analysis Esophagectomy

|  | BS  n=15 | OEP  n=17 | P value |
| --- | --- | --- | --- |
| **Esophagectomy (prim + sec)** | 7 /15 (46.7) | 1/17 (5.9) | .008 |
| Survived | 4/7 (57.1) | 1/1 (100) | .408 |
| Reconstructed | 2/4 (50.0) | 1/1 (100.0) | .171 |
| **Technique of reconstuction** |  |  |  |
| esophagogastrostomy | 1/2 (50) | 0 (.0) | .157 |
| Esophagocolostomy | 1/2 (50) | 1/1 (100.0) | .157 |
| **Outcome of reconstruction** |  |  |  |
| Hospitalisation (range) | 23.00 (21-24) | 18.00 (18.00-18.0) | .317 |
| Clavien Dindo ≥ 3b | 0 (.0) | 0 (.0) | - |
| CCI (range) | 43.60 (43.6-43.6) | 29.60 (29.6-29.6) | .317 |
| survived but not reconstructed * | 2/4 (50.0) | 0/1 (.0) | - |

*, 1x patient in bad physical condition, 1x died due to advanced pancreatic cancer

BS=Boerhaave syndrome, CCI=comprehensive complication Index, OEP=other transmural perforation, prim.=primary, sec.=secondary. Metric data are given in median with range.
